# Developing and evaluating cybersecurity competencies for students in computing programs

**DOI:** 10.7717/peerj-cs.827

**Published:** 2022-01-17

**Authors:** Abdullah Alammari, Osama Sohaib, Sayed Younes

**Affiliations:** 1 Faculty of Education, Curriculums and Teaching Department, Umm Al-Qura University, Makkah, Saudi Arabia; 2 Faculty of Engineering and IT, University of Technology Sydney, Sydney, Australia; 3 College of Education, Al Azhar University, Educational Technology Dept, Cairo, Egypt

**Keywords:** Computer education, Cybersecurity competency, Multi-criteria

## Abstract

Changes due to technological development in the workplace are putting pressure on academia to keep pace with the changing nature of work. Due to the growing need for cybersecurity professionals, universities improve their cybersecurity programs to develop qualified cybersecurity competencies. The purpose of this study is to validate the cybersecurity knowledge, skills, and abilities (KSAs) competencies of cybersecurity degree programs using a fuzzy linguistic group decision-making method. This study shows that cybersecurity knowledge is essential, along with technical skills and human abilities for cybersecurity professionals.

## Introduction

Information technology professionals lacking cybersecurity competencies can result in considerable financial, information and intellectual property losses for organizations worldwide (Choi, Levy & Hovav, 2013). Draganidis & Mentzas (2006) described competency as distinguishable, obvious, and quantifiable skill, knowledge, ability or/and possessing any other deployment-related attributes (such as behaviour, attitude, physical competence) essential for the execution within a specific context. Cybersecurity competencies are a dynamic combination of knowledge, skills, and abilities (KSAs) (Parrish et al., 2018).

Alavi & Leidner (2001) consider knowledge an acceptable belief by which an individual’s ability to create a practical action method is enhanced. As per Prager, Moran & Sanchez (1997), the power to perform mental and physical acts required by tasks is referred to as ‘ability’. Boyatzis & Kolb (1995) stated that skill is a well-organized and goal-directed behaviour accomplished with effort and learned through practice.

Cybersecurity professionals can significantly prevent cyber threats with the implemented policies (Paulsen et al., 2012). In addition, cybersecurity professionals need to possess an advanced level of combined knowledge, skills, and abilities (KSAs) competencies to establish and implement the tools and technologies (Paulsen et al., 2012; National Institute of Standards and Technology (NIST), 2014). On the other hand, not all IT professionals are cybersecurity experts. IT professionals may not be familiar with the modern concepts and lack expertise in cybersecurity and information technology (IT) (Happ, Melzer & Steffgen, 2016; Hazari, Hargrave & Clenney, 2008).

Cybersecurity competencies of computing professionals continue to be an emerging issue (Behrens, Alberts & Ruefle, 2012; Toth & Klein, 2013). Moreover, there is a strong likelihood of advanced and persistent threats on the organizations, resulting from which the confidential data, valuable resources and critical targets are at risk (Marchetti et al., 2016). In addition, despite technical cybersecurity controls, the IT professionals can negate them because they usually don’t possess cybersecurity competencies (Behrens, Alberts & Ruefle, 2012; Al Neaimi, Ranginya & Lutaaya, 2015). One of the most effective vectors to gain access to a secure system is none. Still, phishing attacks since the IT professionals lack the required cybersecurity competencies to a large extent (Bowen, Devarajan & Stolfo, 2012). Likewise, new vulnerabilities are also expected to introduce new technologies, highlighting the need to evaluate cybersecurity competencies (Pittenger, 2016) consistently and precisely. Therefore, recent studies have exhibited the need to assess skills and competencies (Grus et al., 2016).

Implementing cybersecurity can uplift any organization’s security and financial prosperity (Hoffman & Branlat, 2016). In addition, cybersecurity competencies play a vital role in adherence to the regulations, laws and Constitutional requirements (National Institute of Standards and Technology (NIST), 2014). Behrens, Alberts & Ruefle (2012) developed a Competency Lifecycle Roadmap (CLR) for sustainable cybersecurity competencies. Toth & Klein (2013) suggested that all IT professionals are necessitated to acquire essential cybersecurity competencies. However, the dynamic technological changes in the professional workplace call for changes in academic curriculum and strategies. It is important to note that skilled cyber-specialized personnel are essential for mitigating or combating cyber-attacks targeting critical infrastructure. ABET, Inc (2018) continue to develop new criteria for a cybersecurity degree program. ABET is a not-for-profit international organization that accredits computing and engineering programs.

However, acquiring skills involves a decision-making process. Cybersecurity experts face several challenges related to cyber systems. This represents a multiple criteria decision-making (MCDM) issue in assessing cybersecurity competencies. Cybersecurity risk management and assessment is a multi-criteria decision problem (Ganin et al., 2020). MCDM involves the selection of the most appropriate alternative from among various available options with multiple criteria. Several methods could be used for addressing any MCDM issue; however, the effectiveness of each method may vary. The choice of the best method for addressing any MCDM issue is not straightforward due to the ever-increasing complexity of organizational decision-making. Hence, the incorporation of group decision-making strategies in MCDM yields multi-criteria group decision-making (MCGDM). It allows taking decisions involving all members of the groups (Ma, Lu & Zhang, 2010).

Therefore, individuals must perform decision-making in their day-to-day activities to process qualitative information available in a natural or artificial language. Humans commonly perform linguistic decision-making by computing with words (CW) technique (Martinez, Ruan & Herrera, 2010). Experts have put forward several models for linguistic decision-making. But, the processing of information through the CW technique for linguistic decision-making becomes complex when it comes to group decision-making. This may be attributed to the nature of linguistic modelling and the linguistic computational model involved in the process. Herrera & Martinez (2000) were the first ones to introduce the 2-tuple linguistic model, which is extensively employed for CW during group decision-making. This technique offers the advantages of retention of info throughout the CW processes.

Moreover, it allows decision processes to yield error-free and accurate linguistic outcomes. The effectiveness of the 2-tuple model linguistic model was reported for handling data containing uniform and symmetrical distribution of linguistic expressions (Li et al., 2017; Rodríguez & Martinez, 2013). Various applications are based on the 2-tuple semantic as indicated in earlier research (Li et al., 2017; Rodríguez & Martinez, 2013). This study involves the application of the fuzzy linguistic 2-tuple model. This selection may be attributed to the features offered by this model like fuzzy representation, comprehensive and flexible nature and precise decision-making (Rodríguez & Martinez, 2013).

Therefore, this paper adopted a novel 2-tuple fuzzy linguistic group decision-making TOPSIS method developed by Sohaib et al. (2019) to evaluate the cybersecurity required competencies in higher education IT programs. This study follows the work of Behrens, Alberts & Ruefle (2012), Toth & Klein (2013) and Nilsen (2017), who proposed cybersecurity knowledge, skills, and abilities (KSAs) competencies in detail. Thus, this study evaluates the cybersecurity knowledge, skills, and abilities (KSAs) required for cybersecurity competencies. The research question this study addresses. What are the different cybersecurity KSAs criteria needed to meet the cybersecurity competencies in higher education cybersecurity programs in Saudi Arabia?

The paper is organized as follows. The following section provides the literature review of the key concepts studied. Then the study method is presented, followed by the case study analysis. The results are then presented, followed by a discussion and conclusion.

## Literature Review

### Cybersecurity education models

According to the ACM Joint Task Force on Cybersecurity Education (2017), there is a need to develop a comprehensive curriculum in cybersecurity education. The primary purpose of cybersecurity programs is to equip the future generation with cybersecurity knowledge and experience (ACM/IEEE-CS Joint Task Force on Computing Curricula, 2013; ACM/IEEE-CS Task Group on Information Technology Curricula, 2017; ABET, Inc, 2018).

The US National Institute of Standards and Technology (National Institute of Standards and Technology (NIST), 2017) commenced a framework for cybersecurity workforce through collaborative efforts by the educational sector, public and private sectors. The framework also outlines the knowledge, skill, and ability (KSA) essential for organizations and entities who wish to implement cybersecurity in their workplace. Knowledge, Skills, and Abilities (KSAs) define the attributes and traits necessary for individuals and organizations to deliver the required level of performance. Such characteristics may be evident in expertise, skills and experience, acquired through performance-based learning.

ABET, Inc (2018) has specified the accreditation criteria for undergraduate cybersecurity degree programs in addition to current accreditation criteria specified for computer science, Information Systems (IS) and IT programs. ABET is an American private organization serving as an accreditation board for approving various intermediate education programs in engineering, computing and technology. ABET specifies and modifies the criteria to be fulfilled by university programs. ABET, Inc (2017) and ABET, Inc (2018) has contributed to incorporating cybersecurity in existing programs in Saudi Universities. ABET introduced the Engineering Accreditation Commission (EAC) program criteria and made all cybersecurity engineering programs mandatory to meet the Computing Accreditation Commission (CAC) standards besides other existing requirements. CAC criteria make it compulsory to equip the curriculum with rules and activities that ensure safe computing (ABET, Inc, 2017; Parrish et al., 2018).

However, ABET, Inc (2018) also acknowledge the concept of developing independent cybersecurity programs. The measures issued by ABET, Inc (2018) has made it mandatory for undergraduate programs to incorporate cybersecurity principles. In addition, ACM ACM Joint Task Force on Cybersecurity Education (2017) specified comprehensive curriculum criteria in cybersecurity education.

### Cybersecurity competencies

Envisioning the future of cybersecurity is not a simple task. The current cybersecurity activities have been highlighted in the ACM Joint Task Force on Cybersecurity Education (2017) and Institute of Information Security Professionals (2018). Therefore, every IT degree program must incorporate cybersecurity. Hence, the significance of learning about cybersecurity becomes evident from implementing cybersecurity in multiple disciplines, including science, engineering, and business *etc.* This also implies that some knowledge and experience of cybersecurity is essential for every workplace.

The NIST Cybersecurity Framework contains five synchronized and continuous functions: identity, protect, detect, respond, and recover. These functions are fulfilled by having cybersecurity competencies (National Institute of Standards and Technology (NIST), 2014). Moreover, there should be focused measures of competency assessments; hence, the focus of the evaluation must be the functional or technical competencies (Succar, Sher & Williams, 2013). It is worth mentioning that the competency level needs to be established while evaluating an individual (Garavan & McGuire, 2001). Precisely, the experts may design the competency assessments to determine the superior performance level (expert) or a threshold level (minimum competency) (Shahidi et al., 2015). Therefore, all IT users must acquire cybersecurity necessary competency, a dynamic combination of cybersecurity knowledge, cybersecurity skills, and cybersecurity abilities (Parrish et al., 2018; Nilsen, 2017).

### Knowledge, skills, and abilities (KSAs)

All the combined KSAs formulate competencies, which reveals the performance level of the combined KSAs (Chen et al., 2014). The specific actions needed to complete job tasks are directly associated with the KSAs (Baker, 2013). Therefore, the competency gaps involving additional training will be identified once the KSAs are determined (Chen et al., 2014). Besides discovering competency gaps, Baker (2013) expressed that the measures determining the level of task performance are none other than the KSAs.

Different jobs relate to several KSAs in the context of cybersecurity (Campbell, O’Rourke & Bunting, 2015). Specific jobs require a low level of combined KSAs. In contrast, some need a high level of combined KSAs (Lu et al., 2015). Moreover, KSAs are not certainly exchangeable between job functions or career fields (Conklin, Cline & Roosa, 2014). Hence, while determining cybersecurity KSAs, \an initial set of KSAs for all job functions must be the prime area of concentration (Chen et al., 2014; Conklin, Cline & Roosa, 2014).

### Cybersecurity knowledge

According to Alavi & Leidner (2001), knowledge is defined as a reasonable belief that strengthens an individual’s capacity to initiate suitable action. Knowledge can be split into tacit knowledge and explicit knowledge. According to their explanation, the knowledge that can be taught is known as explicit knowledge. In contrast, the knowledge acquired from experience is not easily exchangeable is referred to as tacit knowledge.

As per Parsons et al. (2014), the following cybersecurity knowledge units were described for the students, namely: incident reporting, email use, Internet use, information handling, password management, mobile computing, strong passwords and social networking site use. At the same time, the following IT professionals cybersecurity knowledge units were recorded by Gross & Rosson (2007), *i.e.,* antivirus software, access control, cybersecurity responsibilities, cyber vulnerabilities, cyber threats, file permissions, email encryption, policy compliance, phishing, privacy and sensitive information *etc.* Another cybersecurity knowledge unit is believed to be password reuse (Ives, Walsh & Schneider, 2004). Table 1 shows cybersecurity knowledge competencies.

**Table 1:** Cybersecurity knowledge competencies.

Cybersecurity knowledge competencies	References
Access control	Gross & Rosson (2007); Ifinedo (2012) Parsons et al. (2014); Dye & Scarfone (2014); Ives, Walsh & Schneider (2004); Nilsen (2017); Safa, Von Solms & Furnell (2016)
Antivirus software	
Cyber threats and vulnerabilities	
Email encryption and use	
File permissions	
Incident reporting	
Information privacy	
Strong password and reuse	
Phishing	
Policy compliance	
Sensitive information	

### Cybersecurity skills

The skills required to avoid a loss to IT infrastructure through the Internet are cybersecurity skills (Carlton & Levy, 2015). Cybersecurity skills can be associated with the required tasks or specific sets of actions (Conklin, Cline & Roosa, 2014). Acquisition of skill to prevent unauthorized access to an IT is critical to ensure access control within an organization. It is realized when the individual controls systems (Gross & Rosson, 2007; Ifinedo, 2012). Unauthorized access to sensitive information can only be reduced through proper access control.

The protection offered by antivirus software can be maximized by gaining skill in antivirus software (Dhepe & Akarte, 2013). The automatic update facility of antivirus software is activated by many organizations (*i.e.,* auto-update configurations). However, organizations can come across the times where the update is required to be facilitated by a cyber-security expert (Dhepe & Akarte, 2013). Students must be well aware of handling an antivirus application, especially when the computer system notifies the user to update the antivirus application (Gross & Rosson, 2007).

The students should know the cookie usage skills because they may have unencrypted sensitive information to track the activity (DISA, 2015). Moreover, the students should be familiar with their Internet browser setting to manage their web cookie storage policy (DISA, 2015). Students must have practical skills in email security (DISA, 2015; Parsons et al., 2014). They must know the skill to configure the Email in such a way that prevents leaking confidential data (DISA, 2015; Parsons et al., 2014). Therefore, students must illustrate how they can control the downloading of malicious items besides carrying out the task of exchanging private information with the help of encryption (Barlow et al., 2013; DISA, 2015). Moreover, digitally signing emails must also be demonstrated to provide added security (DISA, 2015; Foster et al., 2015). In addition, the task of scanning all email attachments before use must also be illustrated (DISA, 2015).

Students must also acknowledge the skills in cybersecurity incident reporting to ensure denial of service to an unauthorized person (Imgraben, Engelbrecht & Choo, 2014; Parsons et al., 2014). Students need to know the personal mistakes required to be reported besides identifying suspicious individuals involved in security breaches (Parsons et al., 2014).

During internet connectivity at the workplace, the students must prevent opening the links to malicious Websites (Carlton & Levy, 2015). Hence, students must responsibly demonstrate that they will not click on malicious pop-up windows (DISA, 2015; Kumar, Chaudhary & Kumar, 2015). In addition, students ought to have skills in preventing such activities, through which the systems become vulnerable to malicious code (Barlow et al., 2013; DISA, 2015). As a result of this malicious code, hackers can access the system/network, corrupt the files, and erase hard drives (DISA, 2015). The worms, viruses, spyware, Trojan horses and scripts are included among the examples of malicious code (DISA, 2015). While most students do freelance works, carry out online studies or perform telework, it is appreciated to have skills in securely operating mobile computing devices (DISA, 2015). The task of locking the mobile computing device when inactive must also be demonstrated by the students (Parsons et al., 2014). Skills required by students are the stoppage of password reuse (Ives, Walsh & Schneider, 2004). Concerning confidential data, the skill to prevent phishing attempts is obligatory for the students (Carlton & Levy, 2015; DISA, 2015; Furnell, Tsaganidi & Phippen, 2008). As exposed in the literature review, students need several cybersecurity skills (Table 2).

**Table 2:** Cybersecurity skills competencies.

Cybersecurity skills competencies	References
Preventing unauthorized access	Gross & Rosson (2007); Ifinedo (2012) Parsons et al. (2014); DISA (2015); Deshpande et al. (2015) Carlton et al. (2015); Nilsen (2017);
Using an antivirus application	
Managing cookie settings and usage	
Using incident reporting	
Avoiding suspicious and malicious sites	
Securely operating mobile devices	
Using unique passwords	
Avoiding a phishing attempt	
Securely using social networking sites	
Physically protecting information systems	
Using encryption	
Creating strong passwords	

### Cybersecurity abilities

The physical or/and mental ability to apply skills to execute a task is called the ability (Tobey, 2015). Likewise, the foundation for skills and knowledge application is the ability (Prager, Moran & Sanchez, 1997). The primary cybersecurity abilities are written communication, near vision, advanced written comprehension, written expression and problem sensitivity (Campbell, O’Rourke & Bunting, 2015; Trippe et al., 2014) Nilsen, 2017). The close-up viewing defined for objects almost sixty centimetres or less than two feet from the eyes is described as near-vision or accurate near vision (Colman, 2015). Researchers believe that a cybersecurity ability to view computer screens also corresponds to the near vision concept. According to them, the ability to express when something goes wrong or is likely to go wrong is known as problem sensitivity (Nilsen, 2017). It is nothing to do with problem-solving. Instead, it is only to identify a problem (Trippe et al., 2014).

The ability to read and comprehend government or/and technical documents refers to advanced written comprehension (Trippe et al., 2014). Researchers recommend advanced written understanding as one of the cybersecurity abilities, which can read cybersecurity policies and guidance. The broadcast of a message in written symbols is known as written communication (Terkan, 2013, p. 149). According to Poteet (1980), a visible representation of feelings, thoughts, and ideas is described as written expression, where the writer’s language symbols are used to record or communicate. The experts conclude that written expression is guided as cybersecurity ability. The potential investigators could transcribe cybersecurity incident reports besides speaking to a cybersecurity contact for the relevant issues.

Siponen, Mahmood & Pahnila (2014) support the argument that an entry-level step to follow and implement cybersecurity procedures and policies is the ability to understand cybersecurity terminology. According to the findings of Hagen & Albrechtsen (2009), the ability to anticipate, monitor and respond to cybersecurity challenges are the three fundamental abilities, such as awareness, knowledge and behaviour are needed to measure the information security intervention. Table 3 shows cybersecurity abilities criteria.

**Table 3:** Cybersecurity abilities competencies.

Cybersecurity abilities	References
Oral comprehension	Campbell, O’Rourke & Bunting (2015); Trippe et al. (2014); Nilsen (2017);
Near vision	
Problem sensitivity	
Written communication	
Written expression	

### Fuzzy linguistic decision-making methods

Acquiring knowledge, skills, and abilities involves a decision-making process. According to Ganin et al. (2020), cybersecurity risk management and assessment is a multi-criteria decision problem. Experts have proposed several techniques for complex decision-making issues in the practical world, particularly those involving multiple choices. The selection of a specific method depends on the nature of info available to decision-making individuals. Dominance technique proves effective in case of lack of information available to decision-makers while maximin or maximax technique is used when pessimistic or optimistic information is available.

Moreover, the selection of the method is further categorized into sub-categories in case of the availability of attributes. The conjunctive and disjunctive methods are usually employed if a standard level of information relevant to each attribute is available. However, simple additive weighting (SAW), analytic hierarchy process (AHP), TOPSIS (a technique for order preference by similarity to ideal solution) and the ELECTRE (elimination and choice expressing the reality) method *etc.* are used when ordinal or cardinal scales are used in the analysis of attribute weights (Lu et al., 2007). Although various MCDM techniques are available, the TOPSIS method has been extensively used by previous researchers (Sohaib et al., 2019). The main features offered by TOPSIS that make it favourable are that it is based on the logic of an individual’s preference; moreover, it makes use of simple computation processes and considers the ideal and the anti-ideal solutions concurrently (Shih, Shyur & Lee, 2007). TOPSIS has made it possible to consider criteria and alternatives together (the situation in our study), which is not the case with pairwise comparison techniques. Besides these benefits and the extensive popularity of TOPSIS, it has been our preference because of the features of extensions applicable in fuzzy environments. In this proposal, we will highlight and demonstrate the effectiveness of this feature concerning the ranking of alternatives.

The central underlying concept behind the TOPSIS method is that an alternative is deemed the best one. It needs to correspond to the ideal positive solution and to be contrary to the negative ideal solution besides adhering to the subsequently mentioned steps (Hwang & Yoon, 1981).

### Fuzzy group decision-making methods

Due to the complex nature of the issues faced in the practical world, it is imperative to consider various viewpoints when solving a case. Usually, a group of experts give their opinion, which serves as the solution to the concerned problem; the process is commonly known as multi-criteria group decision making (MCGDM). Previously, group decision-making involved the participation of multiple experts $E= \left\{ {e}_{1},{e}_{2},\ldots ,{e}_{k} \right\} (k\geq 2)$ who present their opinion regarding alternative *X* to solve the issue at hand. The alternative was chosen in two steps, including the aggregation and exploitation stages. The aggregation stage involves using an aggregation operator to compile all the views expressed by different experts. The aggregation operator merges all this data to develop a collective preference matrix containing all the proposed solutions to the concerned problem. This is followed by the exploitation stage, which involves analyzing the joint preference matrix and choosing the most appropriate alternative from all the available options proposed for solving the problem (Rodrıguez, & Martinez, 2013). Sohaib et al. (2019) explain the use of a general scheme which is only possible when experts give their preference from among various available alternatives through the employment of fuzzy linguistic variables. The steps mentioned below are involved in the development of solution scheme (Rodrıguez, & Martinez, 2013):

• The first step involves the selection of a set of linguistic terms that contains semantics. Consequently, linguistics implies meaning and is deemed linguistic descriptors, which experts analyze to determine their preferences from various available alternatives and assign weights to criteria weights in light of their knowledge and experience.
• The subsequent step involves selecting an aggregation operator that compiles all the preferences given by individual experts to present collective linguistic information.
• The final step involves the selection of the best alternative(s) among the available ones.

The aggregation phase involves the application of various models of linguistic computing, including the models proposed by Degani & Bortolan, (1988), Delgado, Verdegay & Vila (1993), Herrera & Martinez (2000). On the other hand, the exploitation phase involves the application of conventional MCDM methods.

### A 2-Tuple fuzzy linguistic group TOPSIS model

TOPSIS techniques and the fuzzy extensions offered by this technique are known for their extensive use in various applications (Ju, Wang & You, 2015; Lu et al., 2016; Wei, 2010). However, this technique fails to meet the computing with words (CW) criteria due to the inaccuracy of the linguistic domain or non-linguistic outcomes indicated by applying the domain of the preferences for distances instead of the linguistic domain. Hence, this drawback called for proposing a novel linguistic TOPSIS model. Such a model is presented in this study. Such a model uses a 2-tuple linguistic model wherein the fuzzy linguistic variables are used to assign weightings to each criterion. Thus, this proposed model satisfies the CW criteria since it involves the use of appropriate syntax and semantics for preferences and distances, leading to precise, flexible and understandable linguistic outcomes.

Let’s consider a set of alternatives *A* = *A*_1_, *A*_2_, …, *A*_*m*_ for the criteria *C* = *C*_1_, *C*_2_, …, *C*_*n*_ being evaluated by a set of decision-makers *D* = *D*_1_, *D*_2_, …, *D*_*k*_. Moreover, considering that the set of the linguistic term for assigning weightage to criteria is *U* = *u*_1_, *u*_2_, …, *u*_*p*_ andconsidering that the set of the linguistic term for the analysis of alternatives is *S* = *s*_1_, *s*_2_, …, *s*_*t*_. Also, consider that the set of the linguistic term to determine the similarity of a linguistic term *s*_*p*_ with another linguistic term *s*_*r*_be *S*′ = *l*_1_, *l*_2_, …, *l*_*t*′_ and the set of the linguistic term to determine the distance of the linguistic term *s*_*p*_ from another linguistic term (*s*_*r*_) *isS*^′′^ = *r*_1_, *r*_2_, …, *r*_*t*^′′^_. Let’s consider a weight vector ${U}_{t}={ \left( {u}_{j}^{t} \right) }_{1\ast n}^{T}$for which the decision-maker *D*_*t*_ ∈ *D* proposes a linguistic value preference ${u}_{j}^{t}\in U$ concerning criteria *C*_*j*_ ∈ *C*. Moreover, consider the decision matrix ${X}_{t}={ \left( {r}_{ij}^{t} \right) }_{m\ast n}$ for which the proposed linguistic value preference is ${r}_{ij}^{t}\in S$ as per the decision-maker *D*_*t*_ ∈ *D* considering the alternative *A*_*i*_ ∈ *A* and the criteria *C*_*j*_ ∈ *C*. In such a case, all decision-makers are supposed to carry an equal level of significance. The steps involved in the advanced version of TOPSIS have been mentioned subsequently. Please see Sohaib et al. (2019) previous work on a novel 2-tuple fuzzy TOPSIS method discussed in detail.

## Method

**Figure 1:**
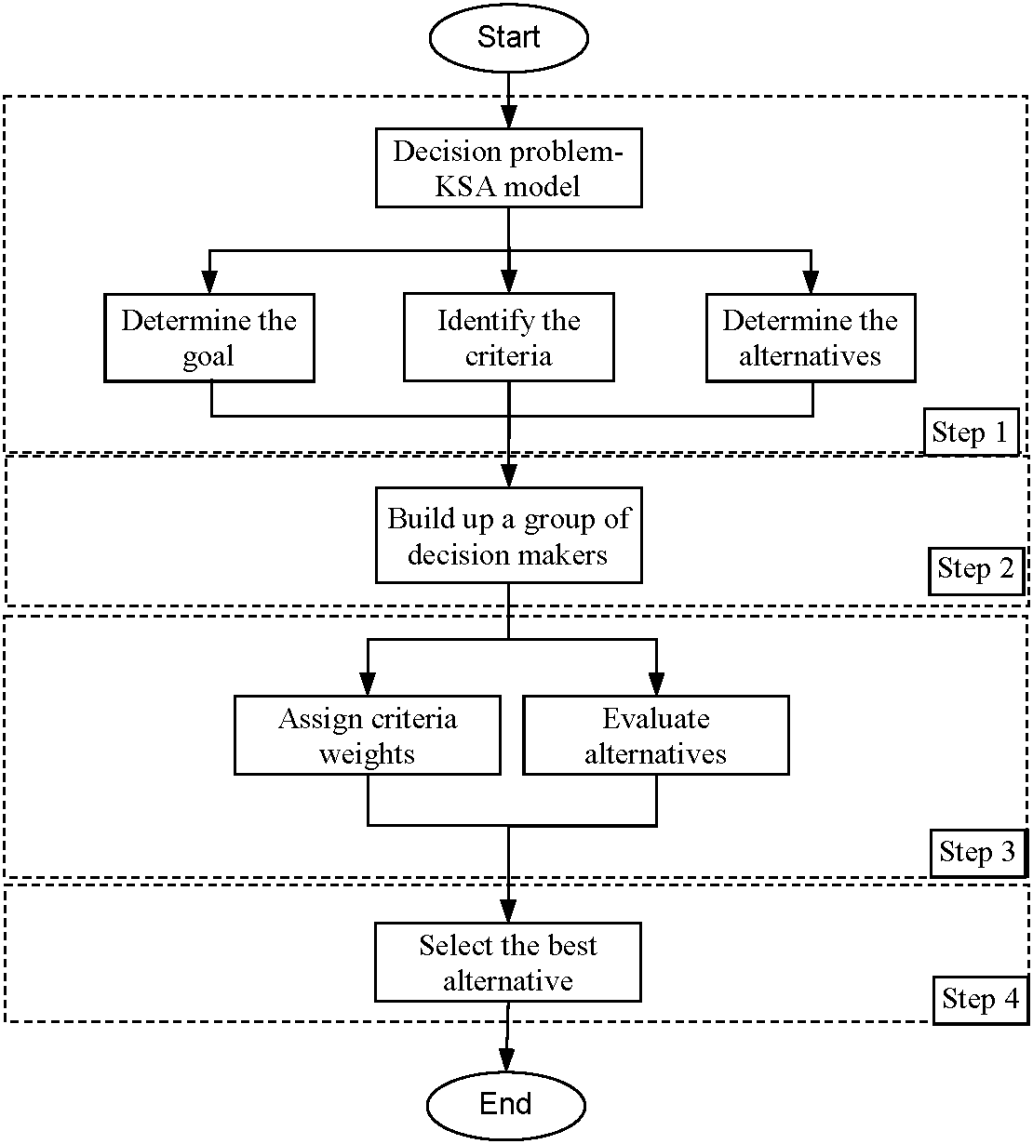
The proposed methodology.

Figure 1 shows the proposed methodology consists of the following four steps.

### Step 1

This research aimed to evaluate the cybersecurity KSAs competencies for computing students of Saudi higher education institutions. The KSA model was used to measure a core set of required cybersecurity knowledge, skills, and abilities (KSAs) essential as cybersecurity competencies (Nilsen, 2017). The three criteria and twenty-seven sub-criteria were determined based on the KSA model. The alternatives are University A (Uni. A), University B (Uni. B) and University C (Uni C.). The criteria structure of the KSA competencies is discussed in the above sections.

### Step 2

In the second step, three professors from three different universities attempted to collectively evaluate cybersecurity knowledge, skills, and abilities essential as cybersecurity competencies. A Delphi method using a ’consensus rule’ was used to improve the process of decision making. Delphi method consensus rule in fuzzy group decision-making aims at making a mutual agreement about an opinion (Lu et al., 2007). Thus, a consensus rule was approved using a questionnaire to build interdisciplinary understanding about the different views.

### Step 3

In step 3, the relative importance of criteria and the alternatives under study was weighted using linguistic terms. The linguistic terms for weighting the criteria and the alternatives are presented in Tables 4 and 5. Triangular fuzzy numbers denote the membership functions of all linguistic terms for the sake of simplicity. Finally, Table 6 shows the linguistic variables for measuring the distance to choose the best alternative.

**Table 4:** Linguistic terms for criteria weighting the criteria.

Symbol	Linguistic term	Fuzzy number
u_1_	Very low (VL)	(0, 0, 0.1)
u_2_	Low (L)	(0, 0.1, 0.3)
u_3_	Medium low (ML)	(0.1, 0.3, 0.5)
u_4_	Medium (M)	(0.3, 0.5, 0.7)
u_5_	Medium high (MH)	(0.5, 0.7, 0.9)
u_6_	High (H)	(0.7, 0.9, 1.0)
u_7_	Very high (VH)	(0.9, 1.0, 1.0)

**Table 5:** Linguistic terms for rating the alternatives.

Symbol	Linguistic term	Fuzzy number
s_1_	Very poor (VP)	(0, 0, 1)
s_2_	Poor (P)	(0, 1, 3)
s_3_	Medium poor (MP)	(1, 3, 5)
s_4_	Fair (F)	(3, 5, 7)
s_5_	Medium good (MG)	(5, 7, 9)
s_6_	Good (G)	(7, 9, 10)
s_7_	Very good (VG)	(9, 10, 10)

**Table 6:** Linguistic terms for calculating the distance.

Symbol	Linguistic term	Fuzzy number
r_1_	Equal	(0, 0, 1)
r_2_	Almost equal	(0, 1, 3)
r_3_	A bit close	(1, 3, 5)
r_4_	Neither close nor far	(3, 5, 7)
r_5_	A bit far	(5, 7, 9)
r_6_	Far	(7, 9, 10)
r_7_	Far away	(9, 10, 10)

### Step 4

Finally, in step 4, the novel 2-tuple group TOPSIS method (Sohaib et al., 2019) was used to obtain the desired ranking. Out of the three alternatives, the best alternative was selected as the ideal strategy based on the maximum closeness degree to the ideal solution.

## Case Analysis and Implementation

As discussed in the research methodology section, cybersecurity competencies criteria in higher education programs in Saudi Universities were evaluated. Three professors teaching cybersecurity from three different institutions were invited to rank three alternatives (Uni. A, Uni. B, and Uni. C) of required cybersecurity knowledge, skills, and cybersecurity abilities vital to cybersecurity competencies. The name of the universities and the participants are preserved to maintain confidentiality. All three universities are based in Saudi Arabia. All experts have more than ten years of industry experience.

### Criteria weights

The criteria weights and the linguistic terms as presented in Table 4; the experts’ judgments have resulted in Table 7.

**Table 7:** Criteria weight matrix.

**Criteria**	**Sub-criteria**	**DM1**	**DM2**	**DM3**
Knowledge	Access control	VH	VH	H
	Antivirus software	MH	MH	M
	Cyber threats and vulnerabilities	VH	VH	VH
	Email encryption and use	H	VH	VH
	File permissions	ML	M	M
	Incident reporting	VH	H	VH
	Information privacy	H	MH	MH
	Strong password and reuse	H	H	H
	Phishing	VH	VH	VH
	Policy compliance	VH	H	VH
	Sensitive information	H	M	M
Skills	Preventing unauthorized access	VH	MH	H
	Using an antivirus application	M	MH	MH
	Managing cookie settings and usage	MH	MH	MH
	Using incident reporting	H	VH	H
	Avoiding suspicious and malicious sites	M	MH	M
	Securely operating mobile devices	MH	H	MH
	Creating and using unique passwords	VH	VH	VH
	Avoiding a phishing attempt	VH	H	VH
	Securely using social networking sites	M	ML	M
	Physically protecting information systems	M	MH	VH
	Using encryption	H	M	M
Abilities	Oral comprehension	M	ML	ML
	Near vision	ML	ML	ML
	Problem sensitivity	L	L	VL
	Written communication	ML	M	M
	Written expression	MH	H	MH

### Alternative evaluation

Table 8 shows an alternative evaluation decision matrix was resulted using linguistic terms (Table 5).

**Table 8:** Alternative evaluation matrix.

Criteria	Sub-criteria	Alternatives	DM1	DM2	DM3
Knowledge	Access control	Uni. A	MG	MG	MG
		Uni. B	G	MG	MG
		Uni. C	VG	MG	MG
	Antivirus software	Uni. A	F	F	F
		Uni. B	MG	MG	G
		Uni. C	VG	VG	G
	Cyber threats and vulnerabilities	Uni. A	VG	G	VG
		Uni. B	VG	VG	VG
		Uni. C	VG	G	G
	Email encryption and use	Uni. A	VG	VG	VG
		Uni. B	MG	G	MG
		Uni. C	F	F	G
	File permissions	Uni. A	P	MP	P
		Uni. B	F	MG	MG
		Uni. C	VG	VG	VG
	Incident reporting	Uni. A	MG	MG	MG
		Uni. B	G	MG	MG
		Uni. C	VG	MG	MG
	Information privacy	Uni. A	F	F	F
		Uni. B	MG	MG	G
		Uni. C	VG	VG	G
	Strong password and reuse	Uni. A	G	G	G
		Uni. B	MG	MG	MG
		Uni. C	MG	F	G
	Phishing	Uni. A	G	G	MG
		Uni. B	MG	MG	G
		Uni. C	G	G	G
	Policy compliance	Uni. A	G	VG	VG
		Uni. B	MG	G	MG
		Uni. C	MP	F	G
	Sensitive information	Uni. A	MG	MP	P
		Uni. B	VG	G	G
		Uni. C	VG	G	G
Skills	Preventing unauthorized access	Uni. A	VG	VG	VG
		Uni. B	MP	MP	P
		Uni. C	F	F	F
	Using an antivirus application	Uni. A	VG	VG	VG
		Uni. B	F	MP	P
		Uni. C	F	F	MP
	Managing cookie settings and usage	Uni. A	VG	VG	G
		Uni. B	MP	P	VP
		Uni. C	MP	P	VP
	Using incident reporting	Uni. A	G	VG	G
		Uni. B	P	P	VG
		Uni. C	MP	MG	MP
	Avoiding suspicious and malicious sites	Uni. A	G	VG	F
		Uni. B	VP	VP	VG
		Uni. C	P	P	MP
	Securely operating mobile devices	Uni. A	G	G	F
		Uni. B	P	VP	VG
		Uni. C	P	P	G
	Using strong and unique passwords	Uni. A	G	G	G
		Uni. B	VG	VG	G
		Uni. C	VG	VG	MG
	Avoiding a phishing attempt	Uni. A	MG	MP	G
		Uni. B	F	F	MG
		Uni. C	G	G	G
	Securely using social networking sites	Uni. A	MG	MG	G
		Uni. B	G	G	VG
		Uni. C	G	MG	MG
	Physically protecting information systems	Uni. A	VG	MG	G
		Uni. B	G	G	P
		Uni. C	MG	MG	MG
	Using encryption	Uni. A	G	G	MG
		Uni. B	VG	G	G
		Uni. C	MG	MG	G
Abilities	Oral comprehension	Uni. A	P	MP	MG
		Uni. B	G	MG	MP
		Uni. C	G	G	F
	Near vision	Uni. A	P	MP	MG
		Uni. B	G	MG	MP
		Uni. C	G	G	F
	Problem sensitivity	Uni. A	MP	MP	P
		Uni. B	VP	VP	VP
		Uni. C	P	VP	P
	Written communication	Uni. A	MG	G	MG
		Uni. B	MP	P	MP
		Uni. C	P	MP	F
	Written expression	Uni. A	G	MG	G
		Uni. B	VP	VP	MP
		Uni. C	P	MP	F

Finally, the novel 2-tuple group TOPSIS method (reference) was applied to deliver a decision as discussed in step 4 of the research methodology. Table 9 shows the 2-tuple linguistic values. The 2-tuple arithmetic mean was to obtain collective values.

**Table 9:** The 2-tuples weights.

Criteria	Sub-criteria	DM1	DM2	DM3
Knowledge	Access control	(u_4_,0)	(u_4_,0)	(u_7_,0)
	Antivirus software	(u_5_,0)	(u_6_,0)	(u_5_,0)
	Cyber threats and vulnerabilities	(u_7_,0)	(u_7_,0)	(u_7_,0)
	Email encryption and use	(u_6_,0)	(u_7_,0)	(u_6_,0)
	File permissions	(u_6_,0)	(u_4_,0)	(u_4_,0)
	Incident reporting	(u_7_,0)	(u_6_,0)	(u_7_,0)
	Information privacy	(u_6_,0)	(u_5_,0)	(u_5_,0)
	Strong password and reuse	(u_6_,0)	(u_6_,0)	(u_6_,0)
	Phishing	(u_6_,0)	(u_7_,0)	(u_7_,0)
	Policy compliance	(u_7_,0)	(u_6_,0)	(u_6_,0)
	Sensitive information	(u_6_,0)	(u_4_,0)	(u_3_,0)
Skills	Preventing unauthorized access	(u_6_,0)	(u_5_,0)	(u_6_,0)
	Using an antivirus software	(u_6_,0)	(u_6_,0)	(u_5_,0)
	Managing cookie settings and usage	(u_6_,0)	(u_6_,0)	(u_5_,0)
	Using incident reporting	(u_7_,0)	(u_5_,0)	(u_7_,0)
	Avoiding suspicious and malicious sites	(u_4_,0)	(u_5_,0)	(u_5_,0)
	Securely operating mobile devices	(u_5_,0)	(u_5_,0)	(u_6_,0)
	Using unique and strong passwords	(u_6_,0)	(u_7_,0)	(u_7_,0)
	Avoiding a phishing attempt	(u_5_,0)	(u_4_,0)	(u_6_,0)
	Securely using social networking sites	(u_5_,0)	(u_5_,0)	(u_3_,0)
	Physically protecting information systems	(u_4_,0)	(u_3_,0)	(u_4_,0)
	Using encryption	(u_7_,0)	(u_4_,0)	(u_6_,0)
		(u_5_,0)	(u_5_,0)	(u_5_,0)
Abilities	Oral comprehension	(u_4_,0)	(u_3_,0)	(u_7_,0)
	Near vision	(u_3_,0)	(u_3_,0)	(u_7_,0)
	Problem sensitivity	(u_2_,0)	(u_2_,0)	(u_4_,0)
	Written communication	(u_4_,0)	(u_6_,0)	(u_4_,0)
	Written expression	(u_2_,0)	(u_4_,0)	(u_2_,0)

Table 10 shows the results of the 2-tuples evaluation matrix and their mean. In the experts’ view, cybersecurity threats and vulnerabilities with a 2-tuple of (u_7_, 0 in Table 9) are the most important criteria with the importance of Very High, followed by access control and policy compliance.

**Table 10:** The aggregated 2-tuples of the decision matrix.

**Criteria**	**Sub-criteria**	**Alternatives**	**Mean**
Knowledge	Access control	Uni. A	(s_7_,-0.2)
		Uni. B	(s_2_,0.4)
		Uni. C	(s_4_,0)
	Antivirus software	Uni. A	(s_7_,-0.2)
		Uni. B	(s_2_,0.2)
		Uni. C	(s_3_,0)
	Cyber vulnerabilities	Uni. A	(s_6_,-0.4)
		Uni. B	(s_5_, 0)
		Uni. C	(s_5_,-0.2)
	Email encryption and use	Uni. A	(s_6_,0.2)
		Uni. B	(s_4_,0)
		Uni. C	(s_5_,-0.2)
	File permissions	Uni. A	(s_6_,0.4)
		Uni. B	(s_6_,-0.4)
		Uni. C	(s_5_,0.4)
	Email encryption and use	Uni. A	(s_2_,0.4)
		Uni. B	(s_5_,0)
		Uni. C	(s_7_,-0.4)
	Incident reporting	Uni. A	(s_5_,0.4)
		Uni. B	(s_6_,0)
		Uni. C	(s_6_,0.2)
	Information privacy	Uni. A	(s_4_,0)
		Uni. B	(s_5_,-0.2)
		Uni. C	(s_6_,0.4)
	Strong password and reuse	Uni. A	(s_6_,-0.4)
		Uni. B	(s_5_,0.4)
		Uni. C	(s_5_,0)
	Phishing	Uni. A	(s_7_,-0.4)
		Uni. B	(s_7_,-0.2)
		Uni. C	(s_2_,0.4)
	Policy compliance	Uni. A	(s_4_,0)
		Uni. B	(s_7_,-0.2)
		Uni. C	(s_2_,0.2)
	Sensitive information	Uni. A	(s_3_,0)
		Uni. B	(s_6_,0.4)
		Uni. C	(s_2_,-0.2)
Skills	Preventing unauthorized access	Uni. A	(s_7_,-0.2)
		Uni. B	(s_2_,0.4)
		Uni. C	(s_4_,0)
	Using an antivirus software	Uni. A	(s_7_,-0.2)
		Uni. B	(s_2_,0.2)
		Uni. C	(s_3_,0)
	Managing cookie settings and usage	Uni. A	(s_6_,0.4)
		Uni. B	(s_2_,-0.2)
		Uni. C	(s_2_,0)
	Managing cookie settings and usage	Uni. A	(s_4_,0)
		Uni. B	(s_5_,-0.2)
		Uni. C	(s_6_,0.4)
	Using incident reporting	Uni. A	(s_6_,-0.4)
		Uni. B	(s_5_,0.4)
		Uni. C	(s_5_,0)
	Avoiding suspicious and malicious sites	Uni. A	(s_6_,-0.2)
		Uni. B	(s_6_,-0.4)
		Uni. C	(s_6_,-0.2)
	Securely operating mobile devices	Uni. A	(s_5_,0)
		Uni. B	(s_7_,-0.4)
		Uni. C	(s_7_,-0.2)
	Using unique and strong passwords	Uni. A	(s_2_,0.4)
		Uni. B	(s_4_,0)
		Uni. C	(s_7_,-0.2)
	Avoiding a phishing attempt	Uni. A	(s_2_,0.2)
		Uni. B	(s_3_,0)
		Uni. C	(s_6_,0.4)
	Securely using social networking sites	Uni. A	(s_2_,-0.2)
		Uni. B	(s_2_,0)
		Uni. C	(s_6_,-0.4)
	Physically protecting information systems	Uni. A	(s_3_,-0.2)
		Uni. B	(s_3_,0)
		Uni. C	(s_6_,-0.2)
	Using encryption	Uni. A	(s_2_,0.2)
		Uni. B	(s_3_,0.4)
		Uni. C	(s_2_,0.2)
Abilities	Oral comprehension	Uni. A	(s_6_,-0.4)
		Uni. B	(s_3_,-0.2)
		Uni. C	(s_3_,0)
	Near vision	Uni. A	(s_6_,-0.2)
		Uni. B	(s_2_,0.2)
		Uni. C	(s_3_,0.4)
	Problem sensitivity	Uni. A	(s_2_,0.2)
		Uni. B	(s_2_,-0.4)
		Uni. C	(s_1_,0.4)
	Written communication	Uni. A	(s_7_,-0.2)
		Uni. B	(s_2_,0.2)
		Uni. C	(s_3_,0)
	Written expression	Uni. A	(s_6_,0.4)
		Uni. B	(s_2_,-0.2)
		Uni. C	(s_2_,0)

## Results

The distance of the alternatives to the negative ideal and the positive ideal solutions were calculated. All the criteria were benefits except problem sensitivity was considered only cost criteria. Table 11 shows the relative closeness degree of each alternative. The results show Uni. B. has the best cybersecurity KSA model as it has a “Far” distance from the anti-ideal solution. There is no need to perform any sensitivity analysis further, as the difference between closeness degrees of alternatives is substantial (Sohaib et al., 2019).

**Table 11:** Alternatives and their closeness degrees.

Alternative	Closeness degree to the negative ideal solution	Linguistic term
Uni. A	(r_5_,0.3)	A bit Far
Uni. B	(r_6_,0.2)	Far
Uni. C	(r_4_,-0.3)	Neither close nor far

The result of this study demonstrates that Uni. B has the ideal cybersecurity knowledge, skill, and ability (KSA) model, followed by Uni. A. Knowledge, Skills, and Abilities (KSA) defines the attributes and traits essential for individuals and organizations to deliver the required level of cybersecurity competencies and performance. Hence, a practical cybersecurity education framework must accommodate all kinds of cybersecurity competencies as defined by KSAs competencies.

## Discussions and Conclusion

Cybersecurity competencies is a dynamic combination of knowledge, skills, and abilities (Parrish et al., 2018; Nilsen, 2017). Cybersecurity competencies focus on performance, which means knowledge alone doesn’t guarantee a successful practising professional in cybersecurity. Technical skills along with human abilities are equally important as knowledge.

Due to the ever-evolving technology and the multidisciplinary field of cyberspace, it has become imperative to develop more comprehensive methodologies and training for equipping future individuals, organizations and institutes with the novel skills and expertise essential for practical implementation of cybersecurity. Cybersecurity is a multidisciplinary field of study covering various legal, human resource, moral and risk management factors. Hence, a helpful cybersecurity education framework needs to accommodate different kinds of competencies.

The implications of this study include providing universities with a validated cybersecurity competencies model for creating cybersecurity assessments. The learning processes for cybersecurity key competencies should attain the three described knowledge, skills, and abilities (KSA) model objectives. The KSA model will also allow students to understand better cybersecurity jobs in a domain that is still undergoing various changes.

Furthermore, the implications of this study include establishing Saudi universities cybersecurity degree programs based on the Knowledge, Skills and Abilities (KSAs) model to meet the accreditation requirements such as ABET, Inc (2017) and ABET, Inc (2018). The US National Institute of Standards and Technology (NIST) (2017), ACM Joint Task Force on Cybersecurity Education (2017) and ABET, Inc (2018) have specified comprehensive criteria in cybersecurity education. It is also imperative for universities and higher education institutes to meet the specified criteria. Such criteria require universities to house a cybersecurity department to offer comprehensive programs with a curriculum covering many mandatory topics related to cybersecurity to equip the students with the Knowledge, Skills and Abilities (KSAs).

A potential limitation of this study includes the use of the Delphi method consists of three experts only. Future work should target in-depth interviews with the industry experts to identify more a comprehensive list of cybersecurity knowledge, skills, and abilities (KSAs).

##  Supplemental Information

10.7717/peerj-cs.827/supp-1 Supplemental Information 1 Questionnaire for ranking

10.7717/peerj-cs.827/supp-2 Supplemental Information 2 Raw data

## Additional Information and Declarations

### Data Availability

The following information was supplied regarding data availability: The raw data is available in the Supplemental File.
