# Supplementary material for: Developing and evaluating cybersecurity competencies for students in computing programs
Source: PeerJ Comput Sci. 2022 Jan 17;8:e827. doi: 10.7717/peerj-cs.827 (PMC8771794; doi:10.7717/peerj-cs.827)
Supplement: Supplemental Information 1 [file peerj-cs-08-827-s001.docx]

**Questionnaire for ranking.**

**Linguistic term**

Very low (VL)

Low (L)

Medium low (ML)

Medium (M)

Medium high (MH)

High (H)

Very high (VH)

| **Criteria** | **Sub-criteria** | **Ranking (Linguistic term)** |
| --- | --- | --- |
| Knowledge | Access control |  |
|  | Antivirus software |  |
|  | Cyber threats and vulnerabilities |  |
|  | Email encryption and use |  |
|  | File permissions |  |
|  | Incident reporting |  |
|  | Information privacy |  |
|  | Strong password and reuse |  |
|  | Phishing |  |
|  | Policy compliance |  |
|  | Sensitive information |  |
| Skills | Preventing unauthorized access |  |
|  | Using an antivirus application |  |
|  | Managing cookie settings and usage |  |
|  | Using incident reporting |  |
|  | Avoiding suspicious and malicious sites |  |
|  | Securely operating mobile devices |  |
|  | Creating and using unique passwords |  |
|  | Avoiding a phishing attempt |  |
|  | Securely using social networking sites |  |
|  | Physically protecting information systems |  |
|  | Using encryption |  |
| Abilities | Oral comprehension |  |
|  | Near vision |  |
|  | Problem sensitivity |  |
|  | Written communication |  |
|  | Written expression |  |
